# Patient preferences for medications to treat opioid use disorder in carceral settings: Implications for tailored implementation and service delivery

**DOI:** 10.1016/j.josat.2026.210099

**Published:** 2026-08-25

**Authors:** Amelia Bailey, Jaclyn M.W. Hughto, Patrick J.A. Kelly, Shira I. Dunsiger, Rosemarie A. Martin

**Affiliations:** a Center for Alcohol and Addiction Studies, Brown University School of Public Health, Providence, RI, 02903, USA; b Department of Behavioral and Social Sciences, Brown University School of Public Health, Providence, RI, 02903, USA; c Center for Health Promotion and Health Equity, Brown University School of Public Health, Providence, RI, 02903, USA; d Department of Epidemiology, Brown University, Providence, RI, 02903, USA; e Department of Psychiatry and Human Behavior, Brown University, Providence, RI, 02903, USA; f Department of Biostatistics, Brown University, Providence, RI, 02903, USA; g Division of Preventive and Behavioral Medicine, UMass Chan Medical School, Worcester, MA, 01655, USA

**Keywords:** Opioid use disorder, Medications to treat opioid use disorder, Incarceration, Criminal legal, Qualitative

## Abstract

**Background:**

A critical lack of knowledge exists about incarcerated individuals' preferences for the provision of medications to treat opioid use disorder (MOUD) in carceral settings (i.e., jail, prison) and of multilevel factors that influence preferences (e.g., health needs, institutional policies). This gap limits the ability to tailor MOUD programs effectively to patient needs and optimize outcomes within correctional systems.

**Methods:**

In 2025, 28 adults receiving MOUD in the community in the Northeastern United States and who previously received MOUD while incarcerated completed an in-depth, semi-structured interview. Transcripts were generated and then coded using template thematic analysis. The social-ecological model informed the interview guide and analysis.

**Results:**

During their most recent incarceration, most participants received methadone (64.3%), one-fourth received sublingual buprenorphine, and the remaining first received buprenorphine then switched to methadone (10.7%). Four themes corresponding to social-ecological levels were developed. First, the context of the carceral environment underlies the MOUD experience while incarcerated, wherein the use of surveillance measures influenced participant experiences of the MOUD program. Second, the delivery of MOUD was affected by logistical complications that influenced MOUD course and could result in participants switching MOUD type or intending to stop MOUD use in the future. Third, MOUD-focused interactions were most often with correctional officers and peers and less often with medical personnel, sometimes resulting in information gaps and perceived stigma magnification. Fourth, withdrawal alleviation and medication maintenance motivated the desire to receive any MOUD while incarcerated, while side effects and knowledge affected the selection of MOUD type. Patients advocated for specific organizational and structural changes to reduce difficulties and enhance comfort and safety (e.g., reducing delays to dosing at intake).

**Conclusions:**

While incarcerated, patient MOUD preferences are informed by several factors spanning social-ecological levels that allow MOUD receipt with more ease (i.e., quicker receipt at intake) and flexibility (i.e., dose changes), and fewer negative interpersonal and individual experiences (i.e., poor interactions, withdrawal, side effects). Patient-guided treatment adaptations have the potential to reach and sustain more patients, advancing equitable provision of MOUD in correctional systems.

## 1. Introduction

Among people with opioid use disorder (OUD), those involved with the criminal legal system (i.e., incarcerated, arrested) face an increased risk of poor health outcomes and disproportionately low access to health services relative to the general population (National Academies of Sciences & Medicine, 2019). Healthcare services provided in jails and prisons are often inconsistent across settings and often fail to meet complex health needs (Puglisi et al., 2017). Immediately after release from incarceration, health risks for these individuals are most severe, resulting in a period of high opioid overdose risk (Binswanger et al., 2013; Pizzicato et al., 2018). Providing medications to treat opioid use disorder (MOUD) for people with OUD while incarcerated is the most effective way to improve health outcomes among this population and reduce the risk of fatal opioid overdose (National Academies of Sciences & Medicine, 2019).

The limited number of carceral-based programs that provide access to MOUD to all individuals with OUD, including methadone, buprenorphine, and/or naltrexone, have shown promise. Use of MOUD during incarceration is associated with reductions in opioid use, recidivism, and mortality, and enhanced MOUD retention upon release (Evans, Wilson, & Friedmann, 2022; Friedmann et al., 2025; Lim et al., 2023; Martin et al., 2023). Despite the benefits of this evidence-based pharmacotherapy, considerable challenges remain to accessing MOUD among people who are incarcerated or who were recently incarcerated (e.g., stigma or fear of discrimination, insurance coverage) (Flanagan Balawajder et al., 2025; Kaplowitz et al., 2023), which reduces access to and overall use of MOUD among people with OUD. In addition, the provision of MOUD can vary substantially across jail and prison settings, as facilities can adopt various models to be able to provide MOUD to individuals (e.g., become a licensed treatment program, transport individuals to an external treatment program); which results in MOUD being provided to patients by trained medical staff either within the carceral facility or external to the facility (American Society of Addiction Medicine, 2025). As the number of jails and prisons in the US offering MOUD or expanding their MOUD eligibility grows (Scott et al., 2021), knowledge of best practices should evolve.

One route to inform MOUD practices is to solicit patient perspectives on MOUD programming during incarceration. The social-ecological model, tailored to the context of MOUD utilization among incarcerated populations, is useful in organizing and understanding such knowledge from patients receiving MOUD (Bronfenbrenner, 1977; Jalali et al., 2020; Russell, Pang, et al., 2022). Using this model, the multivariable and overlapping factors that affect MOUD utilization can be contextualized along individual (i.e., person-level), interpersonal (i.e., interactions with others), organizational (i.e., immediate environment within jail/prison), and structural (i.e., larger social and political context) levels that can collectively shape treatment access, experiences, and outcomes.

Patient perspectives on MOUD utilization during incarceration may be impacted by evolving policies and factors that are specific to the medication. Currently, there are three Food and Drug Administration (FDA) approved MOUD in the US, methadone, buprenorphine, and naltrexone, with extended-release formulations for the latter two. Methadone and buprenorphine are considered the two most effective first-line MOUD (American Society of Addiction Medicine, 2021). Pharmacological and access differences between methadone and buprenorphine impact patient perspectives (National Academies of Sciences & Medicine, 2019; Strang et al., 2020). Preferences toward MOUD types are largely shaped by MOUD program rules, side effects, beliefs about MOUD types, and accessibility and availability of MOUD (Bailey et al., 2025). Most literature on MOUD perceptions and preferences among incarcerated populations was published before significant national developments in the US, such as greater flexibility in MOUD treatment delivery (e.g., removal of X-waiver requirements for buprenorphine prescription, relaxation of take-home policies for methadone provision) and proliferation of fentanyl and other synthetic opioids in the drug supply (Ciccarone, 2021; Krawczyk et al., 2023). As more programs in jails/prisons begin providing all three types of MOUD (methadone, buprenorphine, naltrexone) as well as injectable formulations of buprenorphine (Berk et al., 2025; Scott et al., 2021), it is critical to assess patient MOUD choice. Currently, several domains related to MOUD preferences during incarceration are insufficiently understood: the multifactorial reasons that shape MOUD use during incarceration, whether these reasons differ by MOUD type received, and how these preferences may be impacted by the current era of OUD treatment in 2025 (i.e., one of increased flexibility with complications from the illicit drug supply).

Using a social-ecological lens, we aimed to qualitatively explore MOUD preferences and experiences with MOUD delivery while incarcerated in a facility that provides access to all three types of MOUD. To do so, interviews were conducted with participants released from incarceration (March 2025 – August 2025) to assess experiences with and preferences toward MOUD during incarceration. We examined individual, interpersonal, organizational, and structural factors that inform preferences and decisions to use MOUD during incarceration and MOUD treatment experiences while incarcerated. Findings provide a comprehensive understanding of patient preferences in this context, enhancing the ability to tailor emerging and existing carceral MOUD programs effectively to meet patient needs and improve outcomes.

## 2. Methods

This study was approved by the Brown University Institutional Review Board (IRB). Research was conducted in accordance with the Declaration of Helsinki, and findings are reported in accordance with the COREQ checklist (Tong et al., 2007). Data documentation is published online (Bailey, 2026).

### 2.1. Sample and recruitment

We completed 28 in-depth interviews with people who had received MOUD during a past year incarceration at a Department of Corrections (DOC) in the Northeastern US (one statewide unified jail and prison system located on a single campus). At this DOC, MOUD was provided by a contracted opioid treatment program (OTP) whose staff of counselors and nurses had a presence within the facility to intake, counsel, and administer MOUD. To participate in this study, inclusion criteria were: (1) 18 years or older, (2) English speaking, (3) released from the DOC in the past 12 months, (4) diagnosed with OUD, (5) received methadone or buprenorphine while incarcerated, and (6) willing and able to give informed consent. Individuals were excluded if: (1) currently incarcerated, (2) not willing and able to provide informed consent, or (3) experiencing symptoms of serious psychiatric impairment per judgment of the interviewer (unable to respond to questions about eligibility or study activities, per standardized protocol). Those who received naltrexone during incarceration were not recruited given that few individuals with OUD (<1%) receive naltrexone at this DOC (Martin et al., 2023). Enrollment occurred on a rolling basis beginning in March 2025 using a purposive and convenience sampling strategy. The strategy relied on enhanced recruitment (e.g., tailored materials) of those who might otherwise be underrepresented (e.g., women), and pausing recruitment for over-represented groups (on the basis of gender, race, ethnicity, and MOUD type received while recently incarcerated). Participants were recruited from opioid treatment and recovery providers in the local community within the state where the DOC was located. Recruitment flyers were posted in waiting and exam rooms, and, halfway through the recruitment period, the first author also recruited in-person from clinic sites to purposively recruit underrepresented groups (i.e., those who received buprenorphine while incarcerated) by handing out flyers and discussing eligibility with patients. No participants dropped out.

### 2.2. Data collection

Interested individuals called the study phone line for eligibility screening and, if eligible, the first author arranged an in-person or phone appointment to complete the one time study session. All study sessions were conducted by the first author (female, White, PhD candidate and study investigator), who explained her role in the study and the study purpose during the consent process. The study session included informed consent, brief demographic survey, and a one-on-one interview. Interviews were audio-recorded and transcribed, and averaged 50 min. Participants received $50 for their time (cash or electronic gift card to major retailer).

Following each interview, the first author recorded memos on the interview content and flow, including emerging themes and domains, and areas for interview guide revision. After the completion of *n* = 5 and *n* = 15 interviews, the first author consulted the study team about interview progress and potential subpopulations to purposively recruit. After completion of the 15th interview, the study team decided to stop recruitment of individuals who received methadone only (during their most recent incarceration) and to purposively recruit individuals who received buprenorphine to ensure representation. After completion of the 25th interview, the first author discussed saturation of topics with the study team, determining that responses to interview questions followed similar patterns across participants. Three additional interviews were conducted to confirm that saturation of topics was achieved, and recruitment was stopped.

The verbally administered survey assessed demographics and recent use of treatment services (type received while ever incarcerated, 30 days before incarceration, during incarceration [plus receipt length], and currently). The interview relied on a semi-structured guide to maximize both repeatability and the flexibility essential to collecting rich narrative data (Rubin & Rubin, 2012; Ulin et al., 2012). The guide was iteratively developed and informed by extant literature, the social-ecological model (Bronfenbrenner, 1977; Jalali et al., 2020; Russell, Pang, et al., 2022), and the study team's expertise in qualitative methods. The interview guide questions and probes assessed the following domains: (1) MOUD knowledge and attitudes; (2) preferences and decisions for utilizing specific MOUD; (3) previous experiences using MOUD during incarceration and in the community; (4) intentions to use MOUD during the post-release period, and (5) recommendations for improved MOUD delivery in carceral settings. Within domains, probes identified factors across the social-ecological model, including individual (e.g., internalized stigma, beliefs), interpersonal (e.g., social support, interactions with others), community/organizational (e.g., quality of healthcare, access to medications in local area), and structural (e.g., legal policies, societal stigma) factors that shape MOUD perceptions during incarceration and post-release. After completion of the first 20% of interviews, the guide was revised to improve comprehensibility and add questions for emergent concepts that arose from prior interviews.

### 2.3. Data analysis

Survey data were descriptively analyzed using Stata (StataCorp, 2025). For interview data, transcriptions were created from audio recordings using a HIPAA-compliant transcription service (rev.com, 2025), verified for accuracy, and deidentified by the first author before analysis. Interview data were analyzed using template-style thematic analysis (Braun & Clarke, 2006; Brooks et al., 2015) with deductive and inductive codes. Deductive codes came from the interview guide, and inductive codes were created through open-coding of 3 transcripts by the first and third authors. The first transcript was open-coded line-by-line to generate inductive codes, with the second and third transcripts coded with inductive and deductive codes. The coders met after coding each transcript to discuss code applications; codes were combined, eliminated, or reworded to revise the codebook. Then, an additional 6 transcripts were double-coded using the codebook. Again, the coders met after coding each transcript to review code applications and revise the codebook as necessary. This iterative process enhanced the reliability of the code application. The remaining 80% of transcripts (*n* = 22) were single-coded by the first author, during which code memos were maintained. Data were managed in NVivo (Lumivero, 2022).

Thematic statements were developed by the first author to describe the interrelations between codes (Braun & Clarke, 2006, 2023). To draft themes, the first author first generated a code matrix in NVivo to examine preferences across levels of the social-ecological model (Averill, 2002) and reviewed coding memos to reflect on previous notes on emergent themes (Birks et al., 2008). Throughout this analytic process, data were examined by the type of MOUD used while incarcerated to explore how experiences might differ by MOUD type. Second, the first author examined individual participants' experiences during incarceration and contextualized them within the broader cross-case environment (Miles et al., 2020) to interpret information and identify connections among participants (Crabtree & Miller, 1999). This process included the use of both descriptive and interpretative lenses with this data (Braun & Clarke, 2021). Accordingly, the first author generated themes, revised the themes throughout this process, and consulted with the third author to validate themes. After consultation with the third author, the theme wordings were revised for clarity. After the themes were developed, the first author wrote the narrative, incorporating exemplary quotations. Exemplary quotes are paired with descriptions of the MOUD used by the participant while incarcerated. Participants were not asked to comment on the transcripts or study findings.

## 3. Results

Participants averaged 37.6 years old. Just under half, 42.9% (*n* = 12), were women, with the remaining 57.1% (*n* = 16) men. One participant was American Indian, two were Black or African American (7.1%), and two identified more than one race (7.1%). Two participants identified their ethnicity as Latino (7.1%). Participants were mostly White (82.1%, *n* = 23). In the 30-days before incarceration intake, half were stably housed (50.0%, *n* = 14), with others temporarily housed (14.3%, *n* = 4) or in a residential program (14.3%, *n* = 4). About one-fifth were unhoused (21.4%). Most were unemployed (78.6%), with just over one-fifth reporting any employment just before intake (21.4%).

During their most recent incarceration, 25.0% received only sublingual buprenorphine-naloxone (hereafter referred to as buprenorphine) (*n* = 7), 64.3% received methadone (*n* = 18), and 10.7% received buprenorphine then methadone. Disposition at intake was split, with 53.6% continuing their medication (either methadone or buprenorphine) from the community (*n* = 15), and the remaining 46.4% initiating MOUD during the intake process (*n* = 13). Overall, 86% had *ever* received methadone while incarcerated (*n* = 24), 50% buprenorphine (*n* = 14), and 7% long-acting injectable buprenorphine (LAIB) (*n* = 2). The median length where MOUD was received during the most recent incarceration was 65 days (IQR: 30.5 to 160 days). At the time of the interview, 89% were receiving methadone (*n* = 25) and 11% were receiving buprenorphine (*n* = 3). No participants were receiving LAIB or naltrexone during their most recent incarceration or at the time of interview. Participant characteristics are displayed in Table 1.

Four themes corresponding to social-ecological levels were developed during the analysis: (1) Context of the carceral environment underlies MOUD experience and intensifies perceived MOUD stigma; (2) MOUD delivery affected by logistical complications influenced MOUD course; (3) MOUD-focused interactions with correctional officers (COs) and peers sometimes resulted in information gaps and perceived stigma magnification; and (4) Withdrawal alleviation and medication maintenance motivated the desire to receive any MOUD while incarcerated, while side effects, and knowledge affected selection of type. We describe each theme below.

### 3.1. Theme 1: Structural: Context of the carceral environment underlies MOUD experience and intensifies perceived MOUD stigma

The context of the carceral environment was comprised of patients' perceptions of the prevailing culture of how “they” (i.e., COs, nurses) treated incarcerated individuals: with finite care and surveillance. This culture existed alongside and was supported by policies and practices that conveyed social stigma toward MOUD, ranging from practices undertaken by COs during day-to-day interactions with patients to policies that dictated how MOUD was administered and observed by staff. As one participant described, the context of being in the DOC was the backdrop for receiving MOUD while incarcerated: “The rest [of the things happening within the DOC], of course it's part of [receiving MOUD]. It's all part of one thing.” [1025, White non-Latino man, buprenorphine]. While all participants in this sample remarked on the pervasive culture of the DOC and how it impacted at least some aspect of MOUD delivery, these remarks were more common among those who received buprenorphine while previously incarcerated or during their most recent incarceration. This difference is perhaps due in part to delivery differences between MOUD types - participants described daily dosing for buprenorphine, in contrast to methadone, as stricter and more focused on diversion prevention. Participants acknowledged that buprenorphine, due to the method of administration, is provided differently (i.e., sublingual film) than methadone (i.e., a liquid); given this, buprenorphine is more susceptible to diversion. For daily dosing of buprenorphine, participants described how the entire process was affected by a perceived surveillance via practices to prevent diversion:

“You have the COs in the front of the line that watch you take your medicine, make you lift your tongue up, move your mouth all around. Then after you take your [buprenorphine], you're going to drink a cup of water in front of ‘em. And then they search ‘em out. The gang–their process is just bullshit…And again, they treat you like shit. It's going to go back to they treat you like shit.” [1027, White non-Latina woman, buprenorphine].

Participants receiving methadone also noted diversion prevention practices, like mouth checks, although they did not characterize the daily dosing surveillance with the same intensity or frequency as those receiving buprenorphine. Describing the methadone dosing process and diversion prevention practices, one participant said: “[You] just feel like even more kind of a prisoner… standing there in a single file line against a wall with a CO putting a flashlight down your throat and it just makes you feel dehumanized.” [1015, White non-Latina woman, methadone, previously buprenorphine].

Many participants perceived that the DOC culture had specific negative impacts on patients who were receiving buprenorphine or methadone, relative to incarcerated individuals who did not receive MOUD. Participants shared that they observed some social norms among staff in how staff communicated about MOUD. MOUD-negative statements perpetuated stigmatizing attitudes toward MOUD and were normalized among COs (e.g., statements likening methadone to illicit drug use) and were reportedly disseminated across the institution. As participants shared, these normalized ways of speaking about MOUD within the DOC among staff could result in MOUD avoidance for patients:

“[Methadone dosing] is not a game…we are doing [it] to get help. But the officers…they tell you, ‘oh, you guys that are taking your juice.’ They call [methadone] your juice. ‘Go get your juice’. Or else they call it ‘go get your high’…They say right through the loudspeaker, so everybody knows your business. And that's something that's not cool. You don't do that. That's very disrespectful. You make people feel uncomfortable, make people not want to go do [methadone] anymore. And that's not right.” [1006, Black Latino man, methadone, previously buprenorphine & LAIB].

Participants explicitly identified that social stigma toward MOUD had a role in receipt of treatment while incarcerated. Participant narratives underscore that, while incarcerated and receiving methadone or buprenorphine, they felt unable to advocate for themselves, particularly when encountering stigmatizing comments related to their substance use histories. Such comments were spoken by COs to participants during daily interactions, such as during the daily dosing process. As a participant articulated:

“COs like, ‘you guys, you can go back to the back of the line now. You frickin’ junkie. Can't wait to get your dose’…I was pissed because I was sitting in line and I'm not a junkie. I'm an addict. I was pissed. But we won't say nothing. I'm going to get in trouble. We let it go. At the time I–‘yeah, all right, fine. I'm a junkie.’…No power at all. None.” [1010, White non-Latina woman, methadone].

Across these interactions with staff at the DOC, several participants noted that staff appeared overburdened and, resultingly, more stressed and hurried to complete tasks (like dosing processes). Participants supported DOC in hiring more staff. Patients also advocated for more interfacing with MOUD-educated counseling staff and hiring of staff (i. e., nurses, COs) who were more knowledgeable about MOUD and compassionate generally. With these adaptations, participants anticipated that workload and burnout would be reduced among existing staff. Suggestions for program improvement are included in Table 2.

### 3.2. Theme 2: Organizational: MOUD delivery affected by logistical complications that influenced MOUD course

At intake, all participants reported facing a delay to the first dose of varying lengths (from 24 h to 10 days). Shorter delays (<2 days) led to mild withdrawal symptoms while the longer delays resulted in more severe symptoms and discomfort. Participants described a strong desire to avoid withdrawal symptoms at intake, especially longer or prolonged withdrawal symptoms. A few times, participants recalled not being explicitly screened for prior opioid or MOUD use at intake and advocating for themselves to see OTP personnel (i.e., counselors or nurses whose job is explicitly to support provision of MOUD in the facility) to begin the intake process. After requesting MOUD from OTP personnel, participants speculated delays were caused by communication lags between providers, paperwork snafus, or weekends (i.e., reduced staffing). No rationale was evident to participants for longer delays (>2 days). Longer delays to MOUD dose could impact patient MOUD decisions and intentions. One patient shared he was switching from methadone to buprenorphine in the community before incarceration and, at intake, experienced a severe withdrawal during a 10-day delay until his first dose of buprenorphine. He later switched to methadone because he believed it would be administered sooner at intake in the event he was reincarcerated: “At least 10 days [delay]…I don't know if their dysfunction or if that's their normal process…but it was bad…The methadone's quick…that's why I got back on [methadone].” [1023, White non-Latino man, buprenorphine, previously methadone]. This participant's preference to receive methadone was influenced by a previous experience with delayed medication receipt and withdrawal, demonstrating how organizational delays could alter participants' MOUD decisions.

Participants shared gaps in care that made the intake and MOUD delivery process difficult for them. Sometimes, participants did not receive “comfort medications” (i.e., medication(s) prescribed to reduce withdrawal symptoms, such as ibuprofen) during the intake process, which resulted in their speculation that only certain patients could receive such medications. Others described that they learned to advocate for themselves to receive comfort medications: “It was pulling teeth to get [comfort medications]…so some of [nurses] gave it to you. Though some of them didn't. But you always had to know about it to ask for it.” [1026, Native American non-Latino man, buprenorphine, previously methadone]. Of those who received comfort medications, several found their withdrawal symptoms remained present and uncomfortable.

Participants vocalized experiencing complications when *initiating* MOUD, especially those initiating methadone. Participants shared that initiating methadone required gradually titrating up from 30 mg, with clinical supervision, to find their optimal dose of the medication. After reaching a dose of clinical significance (70-80 mg), participants needed to request additional increases, sometimes leading to a delay. As shared:

“Methadone, [they] only start you at 30 [mg] when you go in and they hold it five days…It takes a while to get to where you feel comfortable. Most people hit like 80 [mg] and start to feel good, and then you can go up depending on your body, your size, your weight…So the first, from 30 to 80 [mg], that five days doesn't really help you…You're still sick. It only holds like two, three hours.” [1023, White non-Latino man, buprenorphine, previously methadone].

Beyond intake, delays in daily dosing were infrequent, with participants often experiencing no barriers to getting to the dosing room/line; participants described this access in contrast to community-based MOUD receipt, highlighting how proximity was a facilitator: “You don't have to drive there yourself, obviously [you're] already in the facility…You just wake up and wait and then you can go in line.” [1009 White non-Latina woman, methadone]. The only notable delay to daily dosing was attributed to an individual's attendance in court for a given day. Participants described how those receiving methadone, traditionally administered mid-day, would not be dosed before court and would receive their dose in the evening. For some, this few-hour delay led to mild distress. To reduce delays in receipt, participants advocated for providing MOUD before leaving for a court hearing and reducing delays at intake.

Participants shared varied preferences for the timing of daily MOUD dosing. The daily dosing of buprenorphine (early morning around 7 am) and methadone (around 12-2 pm) were often not congruent with participants' dose timing in the community. Early dosing, such as with buprenorphine, could result in disruptions to sleep and cellmate relations for most, while some, who liked rising early, appreciated the timing. Alternatively, methadone was perceived as too late in the day by most, incongruent with when dosing is received in the community, resulting in discomfort in the morning as one's dose wanes. Given the timing of dosing before a meal, a few participants noted that receiving a cracker after taking a methadone dose (per dosing protocol) was preferred and helped to settle their stomach. Ultimately, to reduce dosing-related patient difficulties, suggested programmatic recommendations included the daily delivery of MOUD: at times similar to taken/provided in the community (i.e., 8-10 am), with sufficient time to ingest the medication, with enhanced privacy, and with enhanced safety when given to patients with other health conditions (e.g., pain, sleep apnea, heart condition).

During incarceration, buprenorphine was reported to be strictly and quickly administered, negatively shaping MOUD administration experiences. During the daily dosing experience, participants who received buprenorphine often described the dosing process as strict and identified that this was likely due to centering diversion prevention: “[COs] check everything… run [COs] fingers through [a patient's] mouth and they check your hands to make sure you're not trying to put anything in hands. You have to have your hair up, have your sleeves rolled up.” [1007, White non-Latina woman, methadone]. This process differed from methadone, which was often perceived as “pretty simple” compared to buprenorphine [1024, White non-Latino man, buprenorphine then methadone]. Participants felt the administration of buprenorphine was rushed and that this speed undermined the benefits of the buprenorphine. As one participant described, the time where the sublingual medication could dissolve in one's mouth felt reduced and then patients were urged by COs to drink water so that the remaining buprenorphine strip would be fully consumed and non-divertible: “So they put, you get the strips, they rush you through for it to melt in your mouth. You're supposed to melt [buprenorphine] under your tongue. And they're rushing you, telling you to take water and swish it around to get it down…So [buprenorphine] doesn't work the right way.” [1025, White non-Latino man, buprenorphine].

Restrictions on dosage while incarcerated, as perceived by participants, could influence MOUD decisions. Participants recounted that buprenorphine doses were limited to 16 mg once per day for them. For those who desired a higher dose, this perceived limit posed a therapeutic barrier which resulted in them choosing to switch to methadone. A patient with chronic pain described how his buprenorphine dose in the community, 24-28 mg taken over the course of a day, effectively managed his pain and OUD symptoms but the 16 mg received at the DOC did not. This patient elected to switch from buprenorphine to methadone since methadone is a known pain management medication and can be dosed at a therapeutic level while incarcerated. As he described:

“The reason that I switched over was because of the 16mg max of [buprenorphine]. And I was in an excruciating amount of pain…when I was on the outside and I was taking 24 to 28mg, that was a stable dose without me thinking about a pain relief quality from the medication…With methadone, I could go as high or as low as I wanted with the dose, so I switched over to that. Also…they'll prescribe methadone for pain relief.” [1022 White non-Latino man, buprenorphine to methadone].

### 3.3. Theme 3: Interpersonal: MOUD-focused interactions often with COs and peers, sometimes resulting in information gaps and perceived stigma magnification

Interactions with peers could influence MOUD selection and future MOUD use intentions (e.g., taper plan), as conveyed through participants' experiences. One's selection of MOUD type could be altered by peer interactions for several reasons, including witnessing their experiences (e.g., a peer's negative MOUD side effects), learning from them (e. g., discussing “best” MOUD among peers), and altercations with them. One participant in particular faced conflict with a cellmate due to early morning dosing, diversion searches (typically for buprenorphine), and visible withdrawal symptoms which collectively motivated his decision to stop receiving buprenorphine while incarcerated:

“[COs] think everybody trying to sneak [buprenorphine] upstairs. So your cell was always flip and search and it's ridiculous…[COs] popped the door and yell mad early, and [cellmate] doesn't like that. And it was too early for him and it was just too much. And then I'm awake by then at 6 in the morning…You don't eat till 8 or 9[AM]. So I'm just up. And he doesn't like that. So it caused a lot of problems with people. Not everybody's a drug addict, so when you're in the cell pooping your pants and withdrawing, they don't want that.” [1004, Black non-Latino man, methadone, previously buprenorphine].

In addition, while receiving one's dose, participants shared that peers not receiving MOUD would sometimes make judgmental comments to those receiving MOUD. These comments were intensified when dosing was visible to others and, given the time of day and location where dosing occurred, happened most often for methadone. Such comments from peers frequently weighed on participants or led to the desired or attempted concealment of MOUD receipt from their peers, often among those taking methadone, and further reinforced MOUD stigma. Resultingly, participants expressed that patients may conceal receipt by electing to receive buprenorphine because the dosing was less visible or they would tell others they received methadone for pain management, which was perceived to be less stigmatizing. Alternatively, some participants recounted living in a designated unit with *only* others receiving buprenorphine, which they described helped reduce visibility of MOUD and normalized receipt (i.e., no MOUD-related differential among peers where stigma could intensify), thereby enhancing concealment and reducing altercations with others. No participants receiving methadone recalled living in a unit for only individuals receiving methadone, reflecting the jail/prison policies.

“Since they moved everybody into the same block, we all know that everyone takes [buprenorphine]. So it wasn't like when I was on methadone in the past there, when you stand in the methadone line, there's two chow halls next to the methadone line…people are making rude comments about you because of the meds you're taking. With the [buprenorphine], you don't have to deal with that. Not only is nobody really awake…but no one's going to pass to see us.” [1020, Black non-Latino man, buprenorphine, previously methadone].

Participant interactions with medical staff varied and were characterized by participants as brief. Interactions with nurses administering MOUD doses occurred the most often; these interactions were also the most concise and standardized across patients. With other medical personnel, such as OTP personnel (i.e., physician, nurse, counselor), participants expressed appreciation of positive interactions and helpful meetings, though less frequent than desired. To see OTP personnel, patients had to complete a paper request (“med slip”) and wait days for administrative processing until care was received. As a participant described her interactions with counselors and the process of requesting meetings: “You can tell [counselors] really care…You can always put a request slip in…and they call you…It's hard to get to everybody so fast, but it takes a little while sometimes…Probably a few weeks.” [1005, White non-Latina woman, methadone, previously LAIB]. Interactions with OTP personnel supported patient decisions (e.g., met the desire for increased methadone dose). For program improvement, participants recommended more interactions with OTP personnel at intake and throughout their incarceration, including low-barrier, low-touch interactions to check-in on their MOUD receipt.

In contrast to OTP personnel, participants described *frequent* interactions with COs throughout their days while incarcerated. Most participants believed that COs treated all incarcerated persons the same, regardless of OUD diagnosis, race, age, or medical condition: “I've seen these old dude, this old skinhead guy, he's like 80 years old and they just didn't care. They're like, ‘dude, this isn't a hospital.’ So you're an inmate like everybody else…That's their thing. Everybody gets treated the same.” [1004, Black non-Latino man, methadone, previously buprenorphine]. However, when having MOUD-related interactions with COs, many participants described use of stigmatizing language about OUD and substance use: “You know, ‘junkies’…’come on drug addicts.’…[COs] really sucked there.” [1010, White non-Latina woman, methadone]. In this way, the cultural context of the DOC, as identified in Theme 1, was evident through these day-to-day interactions.

Through this use of language, many participants felt that COs did not fundamentally understand that MOUD was an evidence-based treatment. As one participant articulated, these interactions contributed to her wanting to stop receiving MOUD: “[COs] think, ‘oh, they're just on drugs’…I don't think all of them realize that [methadone is] still a treatment…(Interviewer: And did that impact at all how you felt about methadone?). Yeah…I want to get off of it. I don't want to have to keep doing it.” [1005, White non-Latina woman, methadone, previously LAIB]. For others, they were quick to affirm that these comments did not influence their perception of MOUD. No MOUD-supportive interactions with COs were shared. Participants desired fewer interactions with COs that were unsupportive of MOUD receipt.

### 3.4. Theme 4: Individual: Withdrawal alleviation and medication maintenance motivated desire to receive any MOUD while incarcerated, side effects and knowledge affected selection of type

Withdrawal relief was a predominant motivator for receiving methadone or buprenorphine while incarcerated. Depending on the delay to when an individual received their MOUD at intake, withdrawal was either experienced (for longer delays) or anticipated (for shorter delays). A participant described how receiving buprenorphine helped him with such symptoms: “Insomnia, you can't sleep, [buprenorphine] helps with that, it helps with diarrhea, helps with vomiting, stomach pain, abdominal pain” [1026, Native American non-Latino man, buprenorphine, previously methadone] and that this alleviation motivated his desire to receive the medication. A few participants specifically noted that DOC was an undesirable setting to undergo withdrawal, due to the physically uncomfortable setting (i.e., not in own bed/home, without desired foods or drinks) and lack of privacy, reinforcing participants' need for MOUD.

Second to withdrawal relief, participants, primarily those who received methadone, shared that they chose to receive it because “you don't crave drugs” [1008, White non-Latino man, methadone] - effectively reducing cravings and reinforcing their illicit opioid abstinence while incarcerated. A few participants who received methadone specified that they had previously not experienced satisfactory craving reductions with buprenorphine. As a participant articulated, withdrawal symptoms and abstaining from opioids were intertwined, where methadone abated her withdrawal symptoms and desire to use opioids: “Continue staying clean. I wanted to make sure that I wasn't going through withdrawals and getting sick again because I knew that would make me want to do drugs if I was feeling really sick.” [1009, White non-Latina woman, methadone]. For those who continued their MOUD from the community into incarceration, they shared that receiving MOUD during incarceration helped them maintain their recovery or MOUD receipt. Lastly, participants looked forward to the benefits that MOUD could provide when they were eventually released from incarceration. These anticipatory benefits were often centered on maintaining recovery and “not overdosing” [1011, White non-Latino man, methadone].

When participants had experienced past negative side effects with one type of MOUD, they tended to elect to receive the other type while incarcerated. For example, participants expressed choosing methadone when they had experienced side effects they specifically attributed to buprenorphine: teeth decay, hyperactivity, hallucinations, fidgetiness, and stomach upset. Conversely, some shared they simply did not like the way methadone felt and preferred buprenorphine. In addition to side effects, most participants shared lengthy MOUD histories inside and outside of carceral settings where they gleaned knowledge over time that informed their decision on MOUD selection. For individuals naïve to MOUD at incarceration intake, a lack of knowledge about and exposure to MOUD led individuals to make decisions about which MOUD type to receive with minimal information. For example, choosing a type of MOUD based simply on which type others who were incarcerated were receiving: “[The nurse] asked me, which one do I prefer? So at the time I didn't know–I didn't start methadone, I didn't start [buprenorphine] yet, so I said [buprenorphine] because somebody near [me] said they did the same thing.” [1021, White Latino man, buprenorphine to methadone]. Participants desired educational resources and/or consultation on MOUD options for individuals with limited MOUD knowledge at intake.

## 4. Discussion

This study extends knowledge on patient preferences toward receiving MOUD in jail and prison settings, documenting why patients make choices about receiving MOUD and which type to receive. Specifically, we examined the presence of and interaction between factors that influenced this phenomenon residing at multiple social-ecological levels (Bronfenbrenner, 1977; Jalali et al., 2020). At the structural level, participants recognize that the culture of the carceral institution shaped experiences with MOUD while incarcerated through governing how MOUD was provided (organizational-level) and interactions with others (interpersonal). We also investigated differences in experiences and perceptions by the *type* of MOUD received while incarcerated in the past year, revealing how there were some similarities and many differences between receiving methadone or buprenorphine while incarcerated. Lastly, patients recommended adaptations to improve carceral-based MOUD programs based upon their experiences at this carceral facility.

Participants offered different reasons for choosing, or avoiding, methadone and buprenorphine, respectively. Relative to buprenorphine, most participants in this sample preferred methadone as it was provided with greater ease (i.e., quicker receipt at intake, less surveillance when dosed), had more flexibility (i.e., dose changes), and led to fewer side effects in this sample. Importantly, these factors are associated with MOUD preferences and treatment retention: for patients interested in buprenorphine before incarceration, the delivery of buprenorphine in carceral settings, which can be more complex than methadone, can deter patients from selecting buprenorphine across other jail/prison contexts (Russell, Nafeh, et al., 2022). Moreover, patients in this sample described receiving a maximum dose of buprenorphine while incarcerated (16 mg), which, while often therapeutic, is associated with lower retention in community-based samples (>16 mg) (Stringfellow et al., 2025). For these participants who were recently incarcerated, constraints in how buprenorphine is dosed dissuaded their use, and, in turn, may contribute to patient experiences and outcomes upon release into the community (Degenhardt et al., 2023; Nosyk et al., 2024). Paradoxically, buprenorphine is typically accessed with greater ease, relative to methadone, in community settings (Yarborough et al., 2016); raising areas for future research in understanding how patients who receive methadone during incarceration navigate barriers to methadone access post-release. Reducing constraints in buprenorphine provision during incarceration may enhance patient autonomy and support future MOUD engagement. Despite these barriers, participants overwhelmingly described how receiving MOUD of their choice during incarceration benefited their continuous receipt and helped them maintain recovery, echoing extant literature (Bailey et al., 2025; Santelices et al., 2025). Participants' preferences for methadone that were influenced by easier receipt of methadone at this carceral facility, relative to buprenorphine, may not reflect the experiences of patients at other carceral facilities where methadone and buprenorphine are provided under different policies.

Participants shared that the provision of care, often gaps in care (Theme 2), in tandem with individual-level fear of withdrawal from MOUD or illicit opioids, influenced decisions about MOUD while incarcerated. Indeed, participants' fear of withdrawal, paired with experiencing MOUD delays and gaps in comfort medications at intake, seemingly influenced decisions to switch MOUD and future MOUD intentions. Participants advocated for greater low-barrier access to OTP personnel to enhance their MOUD care and reduce experiences of delays or gaps, and withdrawal. While these experiences were highly salient for participants in the carceral setting, some difficult aspects of MOUD receipt may be well reflected in the other contexts of the current treatment landscape. Some participants' experiences while incarcerated were similar to those of patients in community-based samples during the current treatment era, such as heightened fear of withdrawal (Casey et al., 2025; Frank et al., 2023; Frank et al., 2025), and perceived insufficiency of comfort medications during withdrawal (i.e., current medication regimens do not help with symptoms) and of methadone doses during initiation (i.e., starting at 30 mg and slowly titrating upwards allows for more severe withdrawal) (Berisha et al., 2026; Buresh et al., 2022). Medical suites in carceral facilities must navigate the current complexity of managing patient withdrawal (e.g., heightened fear, insufficiency of comfort medications) and balance evolving risks to ensure patient safety (Prescott, 2026). These important factors that patients considered when making MOUD decisions while incarcerated may have been more acute in the carceral setting, where patients solely rely on the facility for medical care (i.e., withdrawal alleviation), which can differ from community settings (i.e., seeking care from multiple providers, use of illicit substances), potentially informing the acuity of their discomfort and desire to avoid such experiences (i.e., prolonged withdrawal) and incarceration in the future.

Participants shared that those *initiating* MOUD experienced lag time to MOUD at intake and also had less knowledge of the system, limiting their ability to advocate for themselves to receive care. Findings align with qualitative research from other carceral systems, showing that delays at intake harm recovery (Santelices et al., 2025) and are worse for those initiating MOUD at intake (Russell, Nafeh, et al., 2022). It is well-established that those who initiate MOUD at incarceration intake, compared to those who had already been receiving MOUD in the community before incarceration and continued receipt at intake, are less likely to stay engaged after release (Krawczyk et al., 2024; Martin et al., 2023). This discontinuity cliff is often attributed to a lack of knowledge/experience with community-based MOUD provision after release. We posit this outcome, wherein those initiating MOUD during incarceration are less likely to continue taking it in the immediate post-release period (Krawczyk et al., 2024; Martin et al., 2023), may be additionally attributed to delays in care at jail/prison intake, which could potentially intensify negative perceptions MOUD. Future research is needed to investigate further. Potential strategies to reduce gaps in care at intake for those initiating MOUD include the use of robust, standardized screening for OUD and dispensing of methadone directly at the jail/prison (National Commission on Correctional Health Care, 2025).

Participants experienced diversion-prevention practices as intensifying surveillance in their day-to-day lives while incarcerated. Diversion of buprenorphine in jails/prisons is indeed a major concern for US carceral facility staff and administration as they seek to ensure the safety of incarcerated persons and staff within facilities (Flanagan Balawajder et al., 2025; Grella et al., 2020). Established carceral MOUD programs rely on diversion policies for the safety of MOUD provision and cite low risk of diversion in their facilities (Evans et al., 2023; Evans, Pivovarova, et al., 2022). Given the experiences of participants in this study, intensive diversion prevention may be harmful for the day-to-day well-being of incarcerated individuals with OUD (i.e., harm to cellmate relations, reduced perceived effectiveness of buprenorphine). While participants undergo similar procedures for all oral medications while incarcerated (i.e., mouth checks) (Tran et al., 2020), the experience of negative interpersonal interactions, as identified by participants in this study, may amplify the perception that buprenorphine and methadone dosing is uniquely stigmatizing. These experiences were typically worse for patients receiving buprenorphine compared to methadone, and therefore, described as a motivator for methadone preference. This finding was endorsed by incarcerated individuals at other facilities, who shared a preference for methadone due to its lower diversion risk (i.e., across facilities methadone is provided as a liquid which is considerably more difficult to divert than oral buprenorphine often administered as sublingual film or tablet) (Evans et al., 2023; Marshall et al., 2023). Ultimately, in the context of the carceral facility, the emphasis on diversion prevention shaped some MOUD decisions. To reduce the potentially negative impact of diversion prevention practices on patient experiences, LAIB may be a viable option for interested patients given its discreet, non-divertible injectable formulation. Indeed, some carceral-based programs prioritize delivering LAIB, over other MOUD, in part to reduce diversion-related concerns (Russell et al., 2026).

Although some participants described interacting with helpful nurses or OTP personnel while incarcerated, most patient accounts emphasized tense interactions between COs and incarcerated persons, which were described as more acute during MOUD-focused interactions. Prior qualitative work has noted interactions with carceral staff that conveyed MOUD stigma to patients impact MOUD experiences in jail (Santelices et al., 2025; Stopka et al., 2024); however, this study is the first to describe the depth of these concerns and how they impact MOUD preferences and future intentions. To enhance patient comfort with DOC staff interactions, participants endorsed increasing all staff numbers to reduce burnout and increasing the number of staff who are compassionate and knowledgeable about OUD/MOUD. Jail administrators across the US have also acknowledged the need for additional staff, for education on OUD/MOUD, and the need to address stigma among staff (Flanagan Balawajder et al., 2025). Stigma toward MOUD that is extended through peers and COs enhances patients' negative perceptions toward MOUD (Santelices et al., 2025), and, in general samples of patients receiving substance use treatment, perceived stigma can deter from treatment continuation (Isman et al., 2025). An overarching strategy to address culture (and stigma) toward OUD/MOUD is to address correctional staff burnout, a phenomenon that often occurs in high-stress settings and can be reduced through intervention (i.e., through stress reduction and efficacy-enhancing strategies implemented across facilities) (Forman-Dolan et al., 2022). Our findings, together with prior work, suggest that it is important to enhance compassion and support toward MOUD in carceral settings.

We identified key factors influencing patient preferences and decisions about MOUD during incarceration and their social-ecological level of influence. In doing so, we identified opportunities to improve the treatment quality and accessibility of MOUD in carceral settings and ways to enhance knowledge, autonomy, and preference-congruence with patient decision-making. While incarcerated, participants focused almost entirely on life within the carceral facility, not outside of it. Given this separation, factors that typically present themselves in one's immediate context regarding MOUD were not notable in the carceral facility (e.g., familial interaction, insurance access) or differed substantially in this setting (e.g., housing) (Bailey et al., 2025; Jalali et al., 2020), thus altering how MOUD use, and barriers and facilitators to it, are conceptualized with the social-ecological model. Additionally, the social-ecological model helped us identify how certain factors were entangled across multiple levels of influence, demonstrating the pervasiveness of these factors, such as stigma toward OUD, MOUD, and incarcerated persons. Indeed, stigma toward MOUD in criminal legal settings impacts institutional support for MOUD and delivery practices (McLean et al., 2025; Pfaff et al., 2024; Rich et al., 2005). Given that stigma-related factors presented themselves across social-ecological levels, they may be especially difficult to address through program adaptation alone. Findings point to a need for multi-pronged service adaptations at structural and organizational levels (e.g., culture, delivery adaptations) to alleviate interpersonal and individual harm (e.g., stigmatizing interactions, internalized stigma) (Komalasari et al., 2021; Kronfli et al., 2025).

### 4.1. Limitations & strengths

There are limitations to this study. This sample was selected to yield context-specific data of experiences at one carceral facility; therefore, findings may not be transferable to other jails/prisons in the US. Additionally, individuals who were not actively receiving MOUD at the time of recruitment may be excluded due to the sampling strategy; future work should explore MOUD perceptions among individuals for whom MOUD is indicated but who declined medication. Findings, therefore, closely inform decision-making processes around MOUD for those receiving it, and future studies should assess the experiences of individuals who received DOC-based MOUD and did not continue on MOUD in the community. In addition, no participants were receiving LAIB or naltrexone at time of interview, urging the need to hear those patient perspectives. Participant insights may also be influenced by recall bias as their incarcerations took place, sometimes, several weeks or months before the interview. Findings may not reflect the current treatment policies and practices at the DOC, as these may have evolved since these prior incarcerations. This analysis also did not examine gender-based differences in MOUD receipt and preferences in this sample, presenting an area for future research. Lastly, this work only represents the patient perspective and future work could benefit from describing the perceptions of DOC staff and administrators. Despite these limitations, this study yielded rich data that may inform data-driven best practices.

### 4.2. Conclusions

Patient preferences for MOUD in jail and prison settings are shaped by interacting individual, interpersonal, organizational, and structural factors. Applying a social-ecological framework, the study demonstrated how institutional culture, which governed daily life and MOUD intake and delivery processes while incarcerated, permeated multiple levels of influence and, per participant accounts, affected access to MOUD and patient decision-making regarding MOUD uptake, type, and post-release continuity plans. Regarding preference for MOUD type, participants we interviewed generally preferred methadone over buprenorphine due to easier access and flexibility, and less diversion-related surveillance. Specifically, negative MOUD experiences while incarcerated (e.g., delays in dosing at intake) undermined perceptions of MOUD benefits and instilled fear (e.g., of withdrawal, of missing doses), especially for those initiating treatment while incarcerated. Overall, findings suggest a need to consider structural and organizational adaptations for MOUD programming in the DOC to improve equitable and effective MOUD delivery in carceral settings.

## Figures and Tables

**Table 1:** Intake characteristics of 28 interviewed individuals who received medications for opioid use disorder during a past-year incarceration.

Characteristic	Total	%
**Age**, years (mean, SD)	37.6	8.7
**Gender**		
Woman	12	42.9
Man	16	57.1
**Race**		
American Indian	1	3.6
Black or African American	2	7.1
More than one race	2	7.1
White	23	82.1
**Ethnicity**, Latino/a	2	7.1
**Disposition at intake**		
Continued	15	53.6
Inducted	13	46.4
**Education status**		
Less than high school	5	17.9
High school or equivalent	13	46.4
Beyond high school	10	35.7
**Housing status at intake**		
Stably housed	14	50.0
Temporary housing	4	14.3
Residential program	4	14.3
Unhoused	6	21.4
**Employment status at intake**		
Employed	6	21.4
Unemployed, looking	12	42.9
Unemployed, not looking	3	10.7
Unemployed, disabled	7	25.0
**During incarceration and post-release**		
**MOUD received during incarceration**		
Buprenorphine	7	25.0
Methadone	18	64.3
Buprenorphine to methadone	3	10.7
**Time incarcerated received MOUD**, days (median, IQR)	65	30.5–160
**Time since release**, days (mean, SD)	154	103.9
**MOUD received currently**		
Buprenorphine	3	10.7
Methadone	25	89.3

MOUD = medications for opioid use disorder (i.e., methadone, buprenorphine, naltrexone); SD = standard deviation; IQR = interquartile range.

Sociodemographic and incarceration characteristics of 28 individuals interviewed in 2025 who received a medication for opioid use disorder (methadone or buprenorphine) during a past-year incarceration at one carceral facility in the Northeastern US.

**Table 2:** Patient participant recommendations to enhance delivery of medications to treat opioid use disorder care while incarcerated.

Social-Ecological Level	Suggestion	Intended Change	Participant Quote
Individual & Organizational (intake)	Implement educational resources or consultation	Improve MOUD knowledge for patients at intake	“Yes, [counseling services] would [help]…For the people to learn about [MOUD] more.” [1018, White non-Latina woman, methadone]
Organizational (intake)	Implement unilateral MOUD screening process	Reduce delays to MOUD at intake	“I think that during the initial screening process that everyone goes through when you first are booked, they go through it with the standard questions before you're even put into a cell. I think that if you need to think you're going to anticipate needing that treatment, that should be asked right then so that they don't go through what I went through, which is like screaming through the cell door that I need the help.” [1015, White non-Latina woman, methadone, previously buprenorphine]
Organizational (daily dosing)	Provide MOUD: At times similar to the community (i.e., methadone in the mornings as it provided at clinics, buprenorphine dosed 2–3 times per day) At a consistent time With sufficient ingestion time (i.e., absorption of buprenorphine) With enhanced privacy With enhanced safety for patients with other health conditions (chronic pain, sleep apnea, heart conditions)	Enhance comfort, effectiveness of MOUD, and safety	“And I just felt like it could have been a lot smoother if we were able to get it earlier…When you wake up, sometimes you don't feel that well. You still feel a little sweaty because the dose only lasts 24 h, so kind of like a shift in your body. So I would definitely change the time.” [1009, White non-Latina woman, methadone]
Organizational (daily dosing)	Provide MOUD before patients leave for a court hearing	Lessen the disruption of some carceral-specific events (i.e., court); enhance comfort and effectiveness of MOUD	“The thing where it sucks, is they wouldn't dose before you go to court because they don't dose you until 5:00 PM on the day that you go to court.” [1013, White non-Latina woman, methadone]
Interpersonal & Organizational (other services)	Greater, low-barrier access to OTP counselors and counseling services (i.e., methadone dose changes, interpersonal support, education, interacting more often with MOUD-educated staff instead of those not (generally trained nurses))	Enhance the quality of MOUD care and reduce negative interactions with other jail staff	“They should have one for [buprenorphine], one for methadone, or just the [MOUD] program in general, where inmates can go and they can talk about their problems and they can meet everybody's needs…the actual counselors and the [MOUD] program people, they could talk to you. You could talk to ‘em. And they can get stuff done way quicker without you getting in trouble.” [1004, Black non-Latino man, methadone, previously buprenorphine]
Structural	More staffing at the DOC	Ensure that supportive staff were accessible and able to conduct their work effectively	“They're really kind of light on the staff. They need more staff. They're so low in COs. They need definitely more staff for medical… And they have to work doubles and triples and quads and they're tired and miserable.” [1005, White non-Latina woman, methadone, previously LAIB]
Structural	Hiring of staff with more compassion and knowledge of MOUD	Reduce the staff's outward stigma toward methadone and buprenorphine	“I just think that they could find more compassionate nurses. You're in the medical field, you take an oath to help people.” [1020, Black non-Latino man, buprenorphine, previously methadone]

MOUD = medications to treat opioid use disorder; CO = correctional officer; DOC = Department of Corrections; OTP = opioid treatment program.

Recommendations provided by 28 interviewed participants on ways to enhance the delivery of medications to treat opioid use disorder care while incarcerated at one carceral facility in the Northeastern US.
